# ADDRESSING AN UNMET NEED FOR MENTAL HEALTH SUPPORT DURING CARE TRANSITIONS FROM NURSING FACILITIES

**DOI:** 10.1093/geroni/igz038.1311

**Published:** 2019-11-08

**Authors:** Kelsey Simons, Katherine Luci, Lauren Hagemann, M Lindsey Jacobs, Emily Bower, Morgan Eichorst, Michelle Hilgeman

**Affiliations:** 1 VISN Center of Excellence for Suicide Prevention, Canandaigua, New York, United States; 2 Salem VA Medical Center, Salem, Virginia, United States; 4 VA Boston Healthcare System, Brockton, Massachusetts, United States; 6 VA Northern Indiana Health Care System, Mishawaka, Indiana, United States; 7 Research & Development Service, Tuscaloosa, Alabama, United States

## Abstract

Mental health (MH) disorders are common among skilled nursing facility (SNF) residents and may inhibit rehabilitation goals. Moreover, discharges to the community from SNFs are periods of heightened suicide risk within the Veterans Health Administration (VHA), suggesting an urgent need for improved continuity of MH care. This paper presents results of medical records reviews indicating a potential gap in MH services at discharge from VHA SNFs. A quality improvement project (”Suicide Awareness for Veterans Exiting Community Living Centers” – SAVE-CLC), designed to address this gap, will also be discussed. Piloted in 3 sites (N = 66) in 2018, SAVE-CLC clinicians administered depression screens by phone to 47 Veterans (71%) after SNF discharge and helped connect Veterans to MH services. 24 Veterans (26%) received a second such call. Patients and caregivers expressed high satisfaction with SAVE-CLC (n = 35, 97%). Implications for quality improvements in SNF care transitions will be discussed.

